# Texturing and Phase Evolution in Ti-6Al-4V: Effect of Electron Beam Melting Process, Powder Re-Using, and HIP Treatment

**DOI:** 10.3390/ma14164473

**Published:** 2021-08-10

**Authors:** Vladimir V. Popov, Mikhail L. Lobanov, Stepan I. Stepanov, Yuanshen Qi, Gary Muller-Kamskii, Elena N. Popova, Alexander Katz-Demyanetz, Artemiy A. Popov

**Affiliations:** 1 Israel Institute of Metals, Technion Research & Development Foundation, Haifa 3200003, Israel; garym@trdf.technion.ac.il (G.M.-K.); kalexand@trdf.technion.ac.il (A.K.-D.); 2 Heat Treatment & Physics of Metals Department, Ural Federal University, 620002 Ekaterinburg, Russia; m.l.lobanov@urfu.ru (M.L.L.); s.i.stepanov@urfu.ru (S.I.S.); a.a.popov@urfu.ru (A.A.P.); 3 Department of Materials Science and Engineering, Technion—Israel Institute of Technology, Haifa 3200003, Israel; yuanshen.qi@gtiit.edu.cn; 4 Department of Materials Science and Engineering, Guangdong Technion—Israel Institute of Technology, Shantou 515063, China; 5 M. N. Miheev Institute of Metal Physics, UB RAS, 18, S. Kovalevskaya Str., 620108 Ekaterinburg, Russia; popova@imp.uran.ru

**Keywords:** electron beam melting, Ti-6Al-4V, EBSD, powder recycling, HIP, texture inheritance, orientation relationship

## Abstract

The research demonstrates microstructural changes and development of specific texture in Ti-6Al-4V specimens produced by electron beam melting (EBM) under different conditions. The effect of two factors, namely, raw material (powder) recycling and hot isostatic pressing (HIP), on the EBM produced samples structure and properties, has been explored. The as-printed and treated samples were investigated using electron backscattered diffraction (EBSD) analysis. Modification of mechanical properties after the EBM and HIP are explained by the EBSD data on microstructural phenomena and phase transformations. The work is devoted to assessing the possibility of reusing the residual titanium alloy powder for the manufacture of titanium components by the combination of EBM and HIP methods.

## 1. Introduction

Ti-6Al-4V alloy is one of the most widely used alloys for metal additive manufacturing by electron beam melting (EBM) and selective laser melting (SLM) techniques. Ti-6Al-4V components have been extensively additively manufactured for industrial applications in the fields of aerospace [1,2,3], biomedical implants [4,5,6], and automotive components [7,8,9,10]. EBM additive manufacturing is recognized as an effective method of producing titanium alloy structures [11,12].

The problem of powder degradation during additive manufacturing is a well-known issue that is being studied by many research groups [13,14,15,16].

There are several key-factors affecting powder during its cyclic re-use:

- Heating factors are attributed to the specificity of EBM process where the powder, surrounding the printed components, during the process is being semi-sintered [17,18];
- Mechanical factors are induced by cleaning and sieving processes before returning the powder to machine for the next printing cycle [14,18];
- Oxidation most crucially affecting microstructure and phase distribution in Ti-Al-4V alloy. Oxidation depends on machine/powder exploitation conditions and on number of printing cycles [13,16,18].

The repeatability of mechanical properties in Ti-6Al-4V components, their density, and final product performance are strongly related to mentioned above issues.

Methods for efficient evaluation and control of mechanical properties in titanium-made parts are in demand by industry. However, mechanical properties testing is relatively high cost and time-consuming [19]. An alternative approach is by investigating the microstructure features of the printed-material and predicting the orientation dependent properties. Moreover, layer-by-layer powder deposition, a high temperature gradient near the melt pool, and directional heat removal can lead to anisotropy of properties as a result of the formation of a morphological and crystallographic texture. Thus, building the relationship between the processing temperature in the manufacturing process and the properties of the printed material can be achieved.

Electron backscattered diffraction (EBSD) based techniques for microstructure analysis and its correlation with final mechanical properties of the examined material, have been previously discussed in [20,21,22,23].

Neikter et al. studied Ti-6Al-4V specimens produced by SLM [20] by using the EBSD technique for reconstruction and description of prior β phase. Their work showed that coarse and fine microstructural areas possessed different parent β phase orientations [20].

Antonysamy et al. studied Ti-6Al-4V alloy specimens build by EBM. They have investigated the effect of build orientation and geometry on the phase distribution and texture [21]. The EBSD measurements were carried out for reconstruction of the prior β phase orientation. The authors showed the effect of geometrical features (build orientation, wall thickness, inclination angle, etc.) on the obtained texture and microstructure [21].

Wang et al. investigated the effect of beam speed on the Vickers hardness and elastic modulus of Ti-6Al-4V specimens produced by EBM process [23]. They concluded that the speed function 50 resulted in highest values of these mechanical characteristics. In this work, EBSD results demonstrated the correlation between highest mechanical properties of the sample with speed function 50, and its microstructural behavior and texture evolution [23].

Ganor et al. [24] and Popov et al. [25] demonstrated positive effect of HIP on fatigue strength of EBM-made Ti-6Al-4V parts. Moreover, Ganor et al. showed that lower temperature HIP is beneficial for this process and this material, which resulted in increased fatigue resistance and elongation [24]. Leon et al. confirmed that HIP treatment for Ti-Al-4V parts built by Arcam EBM process results also in corrosion properties improvement due to increase of β phase amount [26].

The motivation of this work is to utilize the EBSD based methodology for proper understanding of phase transitions and microstructural evolution in EBM-made Ti-6Al-4V and then to determine dependences between microstructural behavior and mechanical performance of the printed components. In this study, the effect of powder degradation/oxidation (due to recycling) and effect of HIP treatment on the phase distribution and texture was investigated.

The additional motivation of the research is the fact of powder ‘aging’ and oxidation while recycling in powder bed fusion (PBF). The recommended procedure is to mix/add the new powder in re-used powder while recycling [27]. We propose an alternative approach to how the severely recycled powder can be effectively used for industrial applications.

Comprehensive data about the manufacturing process and the mechanical properties of the additively manufactured samples (from both powder batches) before and after HIP were presented in [18]. In the previous research, the experimental findings were explained based on the characterization and analysis of the powder morphology and powder oxidation degree. In the current study, we want to show correlation between microstructural parameters, oxygen content, and hardness values after 0 and 69th powder cycle.

The correlation between used powder state, microstructure and texture of the additively manufactured material, and final part quality is still an issue that needs systematic investigation. To contribute to the understanding of the physical dependencies related to powder-bed additive manufacturing and post-processing, the mechanisms and fundamental microstructural features of phase transformation need to be studied and characterized. In the present study, the microstructural characteristics of additively manufactured samples produced under different conditions has been investigated as a function of the number of re-use cycles, oxidation, and heat treatment. The current research is focused on studying the patterns of formation of structure and texture in the process of multiple thermal effects in combination with HIP. Systematic microstructural investigation and analysis of phase distribution and phase formation mechanism is a possible alternative or valuable addition to the high-cost mechanical properties testing in the evaluation or prediction of the mechanical performance of the printed parts.

Table 1 summarizes the previous research on the same topic. It can be seen that only the current study which actually is the follow-up of [18] analyzes the effect of powder degradation on the final microstructure and properties of the EBM-made samples. Moreover, in this work, the EBSD characterization technique is used to representatively show the influence of the powder state and effect of HIP treatment on the phase transformation behavior in Ti-6Al-4V samples. The novelty and the scientific value of the presented study can be formulated as follows:

- Four groups of EBM-made samples produced under different conditions were systematically investigated.
- In one study, we explored both the effect of powder degradation due to repeatable re-use and the effect of HIP treatment on the additively manufactured samples.
- Based on previously obtained mechanical tests results (i.e., ‘macro’ results), we thoroughly investigated the selected four groups of samples by EBSD technique to obtain the microstructure characteristics (i.e., ‘micro’ results).
- We showed the process of phase recrystallization occurring during EBM and HIP, and analyzed texture of solidification and phase transformations formed during primary melting.

## 2. Materials and Methods

All the specimens for the research were manufactured using Arcam EBM A2X (Arcam AB™, Krokslatts fabriker 30, Mondal, Sweden) machine located at Israel Institute of Metals (Haifa, Israel). The commercial Arcam’s gas atomized Ti-6Al-4V powder was utilized as a raw material for the additive manufacturing trials [18]. The manufacturing was conducted in vacuum 10^−2^ mbar; with the standard layer thickness of 50 µm; and using default parameters for Ti-6Al-4V processing (see Table 2). According to the simulation reported in [34], the maximal temperature developed in the upper heated layer was approximately 2000 °C, and then the mentioned certain layer was reheated for at least seven times up to more than 1000 °C due to next heating cycles applied to the next built layers. Effect of such a re-heating regime has been comprehensively discussed in [34]. The effect of powder recycling was investigated by comparing the EBM manufactured components using the virgin powder batch and the one after the 69th printing cycle [18].

**Figure 1:**
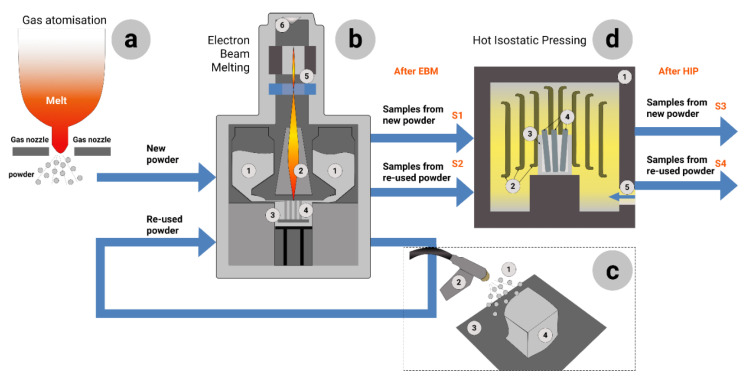
Flow of processing and experiment: (**a**)—Ti-6Al-4V powder gas atomization; (**b**)—electron beam melting, where 1 stands for powder in hoppers; 2—high-power electron beam; 3—building platform; 4—additively manufactured cylindrical samples in powder bed; 5—focus and deflection coils; 6—cathode grid; (**c**)—powder recovery system (PRS) for cyclic use of the powder, where 1 stands for Ti-6Al-4V powder; 2—powder spray gun; 3—building platform; 4—semi-sintered powder bed with samples inside; (**d**)—hot isostatic pressing (HIP), where 1 stands for internal chamber of the HIP furnace; 2—heating elements; 3—container; 4—additively manufactured cylindrical samples.

Figure 1 shows the design of the experiment: the gas atomized Ti-6Al-4V (new powder) used for EBM (Figure 1b); then after EBM the half of the produced samples have been used for S1-samples. Another half of as-built samples passed hot isostatic pressing (HIP) (see Figure 1d) and the S3-samples for characterization were prepared. The same workflow was for re-used powder. The powder that passed 69 cycles of additive manufacturing, has been used to prepare S2-samples (as-built without HIP), and for S4-samples—that were produced from re-used powder and passed HIP treatment.

The used powder for the current research was 80 kg of Ti-6Al-4V (grade 5) powder supplied by GE-Arcam. The first set of samples was additively manufactured from as-received new powder (S1 samples). After finishing the EBM process, the chamber was cooled to nearly 50 °C before opening. The chamber has been cleaned of the powder which was collected by a standard vacuum cleaner for metal powders. The set of additively manufactured samples ‘locked’ inside the sintered powder block was transferred to the powder recovery system (PRS), where it was blasted using the same Ti-6Al-4V powder to release the printed samples (see Figure 1c). The removed sintered powder from the PRS was then mixed with the virgin powder from the hoppers, sieved together, and placed back into the hoppers. The EBM process and powder recovery procedure were repeated for a total of 69 cycles. After the 69th cycle, the second set of samples was additively manufactured (S2 samples) from the highly re-used powder.

As it is shown in Figure 1 and Table 3 there were four groups of samples in this research:

- S1-samples were additively manufactured by EBM from new powder and investigated in as-built state;
- S2-samples were additively manufactured by EBM from re-used powder and investigated in as-built state;
- S3-samples were additively manufactured by EBM from new powder and investigated after HIP treatment;
- S4-samples were additively manufactured by EBM from re-used powder investigated after HIP treatment.

For microstructural characterization and mechanical testing, five samples of each group were prepared.

EBSD measurements were carried out using a Zeiss Ultra Plus scanning electron microscope (SEM) equipped with a Bruker EBSD detector. SEM samples were prepared by grinding with SiC paper up to 2400-grit, followed by polishing with a mixture of 90% colloidal silica (Struers OP-S) and 10% hydrogen peroxide. The EBSD data was used to reconstruct α and β grains with Oxford Instruments software.

For texture analysis, the coordinate system (*X*, *Y*, *Z*) is taken as a laboratory one. In all figures, the *X*-axis was set to be parallel to the horizontal axis of the sample, the *Y*-axis is parallel to the vertical axis of the sample, and *Z*-axis is normal to the plane of the figure, that is, the plane from which the analysis was performed. Moreover, the *Z*-axis coincides with the building direction (BD). The orientations of the texture components in Miller indices were determined with respect to this coordinate system.

The phase identification and orientations analysis were performed for all studied areas with a degree of recognition above 96%. The formation of orientation maps was carried out with the ‘restoration’ of unrecognized elements, using the procedure of fixing single points.

Preliminary studies of misorientation distributions of α-phase grains revealed peaks located at misorientation angles of 10 ± 2°, in addition to the angles smaller than 4°. Because of this feature, orientational maps including high-angle grain boundaries with misorientation angle ≥8°, have been built for analysis of grains morphology. The boundaries thickness on these maps is 1 pixel. Oxford Instruments software was used for a definition of average size (d_α_) of α-phase crystallites, which was calculated as a diameter of a circle equivalent to a mean crystallite area (see Table 3). The crystallites with misorientation angles of more than 8° have been taken into account.

The amount of β-phase (see Table 3) in all the investigated areas were of a small (2, ..., 5%) fraction, but sufficient for reliable identification. A module of the Oxford Instruments software was involved in the work, which makes it possible to analyze the presence and accuracy of the specified orientation relationships (OR) between the β- and α-phases and to specify the distributions of the interphase boundaries at the angles of their deviation from the ideal. In the analysis, the Burgers OR was set to be: {1–10} <111> β‖ {0001} <11–20> α. On the phase maps, the β-phase regions are identified in blue.

**Figure 2:**
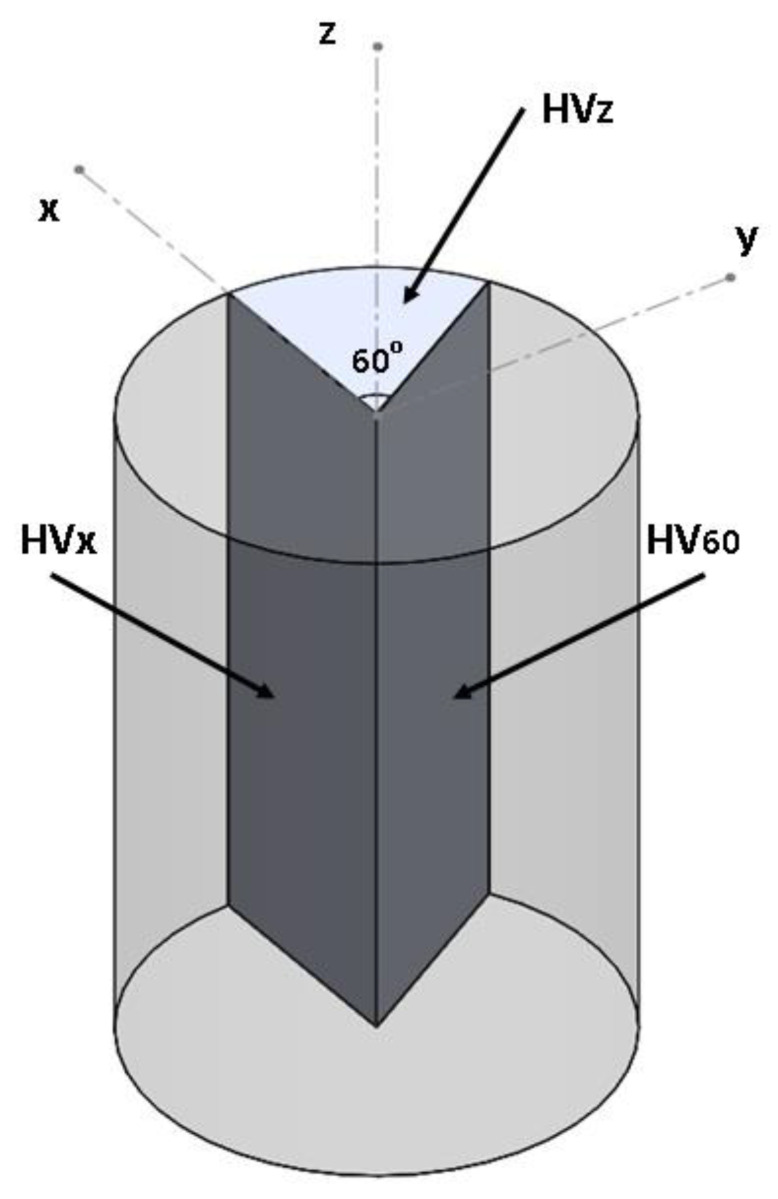
Samples cutting for microhardness test.

For the hardness test samples have been cut in three planes as it is shown in Figure 2. Samples were mounted in Bakelite and polished using SiC abrasive papers. Samples were not etched. Microhardness has been measured using Microhardness tester Future-Tech FM-110.

## 3. Results

**Figure 3:**
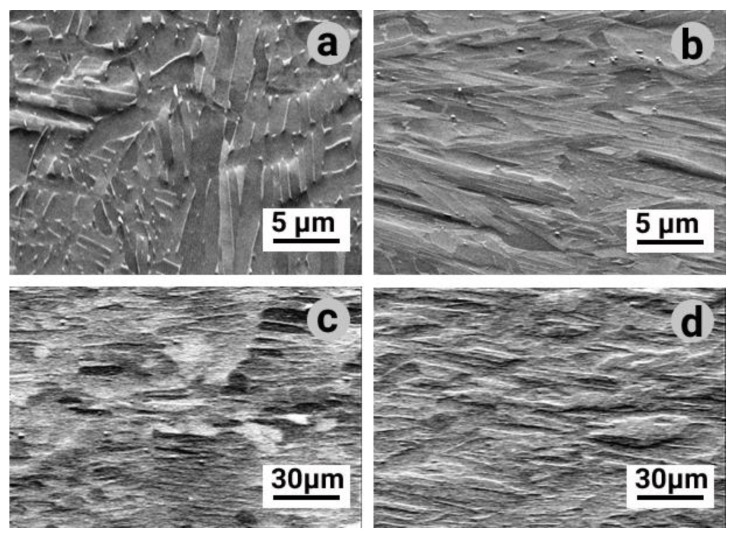
SEM micrographs of EBM manufactured Ti-6Al-4V samples with and without the HIP process: (**a**) S1, (**b**) S2, (**c**) S3, (**d**) S4.

**Figure 4:**
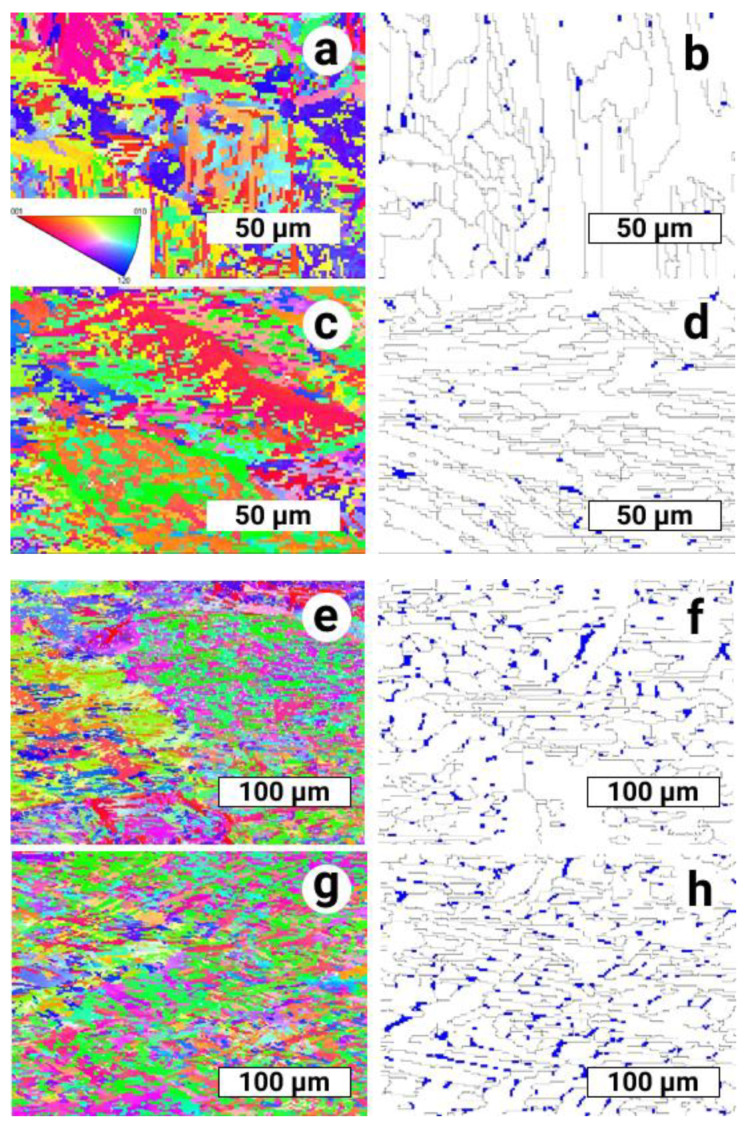
Microstructure of the alloy after EBM and HIP in the form of orientation maps (EBSD): Left column—of α-phase maps in Z-direction with standard color key; Right column—phase map with α-phase (white), interphase and intergranular boundaries (black lines), β-phase is indicated (blue); (**a**,**b**) S1; (**c**,**d**) S2; (**e**,**f**) S3; (**g**,**h**) S4.

The microstructure of all EBM-built samples without and with a post-HIP process revealed typical α-phase lamellar structure (see Figure 3 and Figure 4), which is in agreement with the results in [24,25,26]. Except from these lamellae, some amount of larger α-phase grains exhibited more equiaxed shape (Figure 4b,d,f,h). The presence of the latter indicates the partial recrystallization of the α-phase during in heat affected zone. In combination with the slightly varying average crystal size of the α-phase (dα, Table 3) after HIP in comparison with EBM without HIP, only a weak development of recrystallization processes can be assumed.

**Figure 5:**
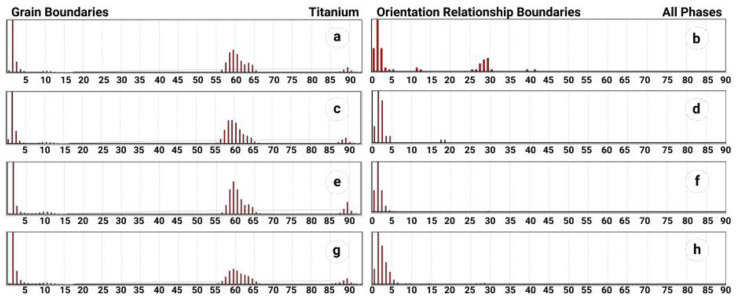
Spectra of grain boundaries by misorientation in the α-phase (left column) and deviations of interphase boundaries from Burgers OR (right column): (**a**,**b**)—S1; (**c**,**d**)—S2; (**e**,**f**)—S3; (**g**,**h**)—S4.

Lamellae of the same colony are divided mainly by high-angle grain boundaries close to 60° (see Figure 5a,c,e,g). In addition, some strong peaks corresponding to 90° are clearly distinguished in the spectra of misorientation distribution. A weak maximum can also be distinguished in the vicinity of 10 ± 2° (Figure 5a,c,e,g). Such a definite misorientation angles distribution evidences about their common crystallographic genesis, which resulted from phase transition with a pronounced displacive component [35,36].

The β-phase were mainly located on the inter-lamellar grain boundaries in a shape of ‘fine’ (two pixels) as well as relatively ‘coarse’ precipitates (Figure 4b,d,f,h). Small (but distinguishable) amount of β-phase was revealed inside some lamellae and coarse α-phase grains (Figure 4f,h). Appearance of ‘chains’ of such precipitates, which are not connected to the inter-lamellar boundaries, can be met quite often. Existence of such β-phase ‘chains’ inside the coarsest α-grains permits suggesting β-phase precipitation onto inter-crystalline boundaries of α-phase, followed by their further coagulation and partial recrystallization progress in α-phase by a normal grain growth mechanism.

**Figure 6:**
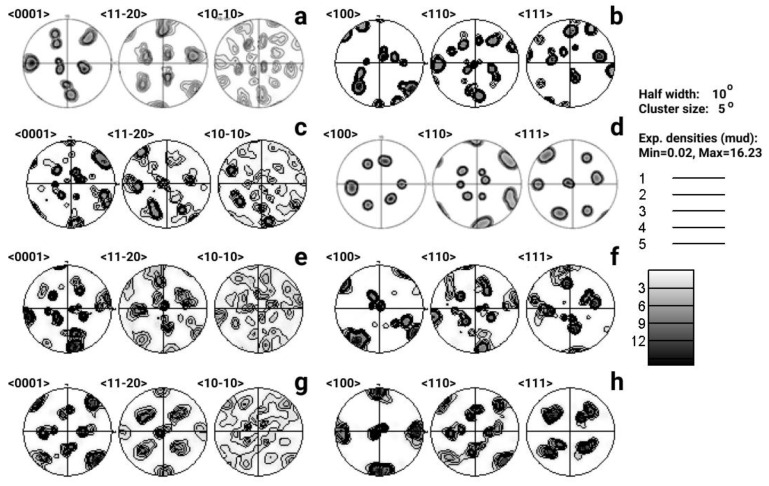
Pole figures <0001>, <11-20>, and <10-10> for α-phase (left column) and <100>, <110>, and <111> for β-phase (right column): (**a**,**b**) S1; (**c**,**d**) S2; (**e**,**f**) S3; (**g**,**h**) S4.

It is important to emphasize that the texture analysis made for the two mentioned phases of all studied zones (in all samples) has been performed for a small amount of parent β-phase—1–3 grains (Figure 6). Therefore, such analysis gives only rough estimation of the integral crystallographic structure of the whole EBM coupon.

According to pole figure (PF) analysis (Figure 6b,d,f,h), crystallographic texture of β-phase in all studied zones is represented by 1–3 grain orientations, which are quite close one to another. Axis <001> of these orientations is close (almost parallel) to *Z*-axis. In all studied cases, the crystal texture of α-phase is found to be multi-component. It contains a certain amount of 6 to 12 preferred orientations (amount of the main reflexes on PF (0001) for α-phase Figure 6a,c,e,g). Partial but sufficiently exact coincidence of the main poles on PF <110> β and <0001> α suggests orientational dependence between orientations (Burgers OR) of the phases β and α.

Orientational analysis between β and α phases (Figure 5b,d,f,h) showed that the majority of β-phase demonstrate strict Burgers OR with α-phase crystallites [37,38]. Symmetry axis of Gauss distribution of the spectrum of misorientations is shifted by 1° from ideal BOR, while the deviations do not exceed ±3°. Therefore, one can suggest that major fraction of β-phase precipitates was formed as a result of phase transformation with dominate displacive component.

The microstructure of the Ti-6Al-4V alloy obtained in PBF additive manufacturing is a (α + β) structure formed from columns of a parent β phase. The microstructure of the specimen printed using this technology depends on two main parameters: the initial cooling rate and the temperature gradient that changes due to the number of heating cycles obtained due to the specificity of the EBM technology. The initial cooling rates in the melting pool area in PBF processes are significantly greater than the critical cooling rates for the diffusion transformation in Ti-6Al-4V alloy. Therefore, the α’- martensite phase is most commonly observed in SLM and EBM stratification production processes. At the same time, it is important to note that following a number of thermal cycles during production, this phase can perform beyond the structure of (α + β).

It is known that the microstructure of the Ti-6Al-4V alloy obtained by PBF represents columnar grains of the initial β-phase formed as a result of epitaxial growth [21]. As the molten layer of powder cools, the high-temperature β-phase undergoes a martensitic transformation with the formation of acicular-shaped α’-martensite [20]. In addition, heating the powder layer above the melting temperature leads to the fact that the previously crystallized layers become a heat-affected zone, a defocused electron beam to a temperature of approximately 700 °C heats the powder and the building plate is heated to 750 °C. This leads to multiple phase recrystallization of previously crystallized layers.

The established fact that <001> axes of the β-phase are parallel to *Z*-axis, coinciding with the direction of energy supply, and, accordingly, preferential heat removal, correlates with the well-known fact of the growth of bcc crystals of refractory metals during their solidification in the <001> direction [39]. Moreover, the obtained data are consistent with the results of [22] on the formation of texture in titanium alloys in EBM additive manufacturing. It is generally assumed that according to the Burger’s relationship and the symmetry of α and β phases, a total of 12 distinct α orientations may arise from an initial β orientation during the β → α transformation. Since a single α orientation could result in 6 β-orientations and a single component in β-phase texture can give rise to α-phase 12 components, totally 72 α-orientations can occur with equal probability after α → β → α transformation [39]. This indicates that repeated cycling in PBF leads to a random texture.

However, in a bcc-material that initially had a solidification β-texture and has been repeatedly subjected to heat treatments (repeated heating/cooling during EBM and heating while HIP) the crystallographic texture can be completely rehabilitated when the material passes into the high-temperature bcc-state. Taking this into account allows assuming the realization of structural and textural inheritance, which was repeatedly observed in steels [40,41], both during the Ti-6Al-4V additive manufacturing and during post-HIP processing.

**Figure 7:**
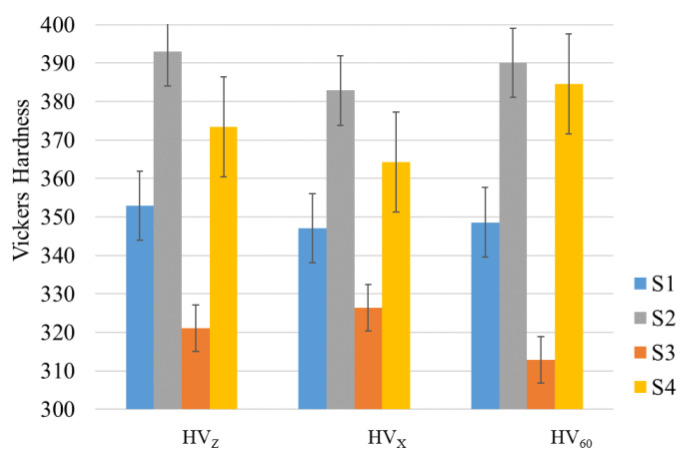
Vickers microhardness results for the four groups of the samples in selected planes.

Microhardness measurements (10 measurements per sample; HV-200g for 10 s) performed on samples cut from different directions (see Figure 7). The results showed almost isotropic values within each specific sample. This can be explained by the multicomponent weak crystallographic texture of the α-phase, with a uniform distribution of the α-lamellae orientations in space (see Figure 6a,c,e,g), which were formed as a result of β-α transformation in accordance with the Burgers OR.

The oxygen content is a crucial parameter in EBM that affects the mechanical performance of Ti-6Al-4V parts. Table 4 shows that powder recycling causes oxygen growth both in powder and in samples built from the reused powder [18].

Since oxygen is a strong α-stabilizing element, and its ability of effectively strengthening α-titanium [18,42], increased oxygen content in the samples built form the recycled powder batch caused increase of their overall microhardness [31,43]. At last, the decrease in microhardness during HIP at a temperature of 920 °C after EBM is associated with the recovery and recrystallization in the α-phase.

## 4. Conclusions

An experimental investigation on four groups of EBM-made Ti-6Al-4V specimens was performed using SEM and EBSD techniques. Samples were manufactured utilizing virgin and re-used (after the 69th cycle) powder batches, in as-built state, and after the post-HIP treatment.

The prior work [18] showed that application of HIP for the titanium components additively manufactured from non-ideal powder radically improves their fatigue resistance [18]. Usage of HIP reduces wastes in additive manufacturing process, increases its industrialization and efficiency [18]. In [18], it was described a negative effect of the powder damaged by numerous recycles on some mechanical properties (mainly, fatigue resistance) of the as-built product. However, the mechanisms of such influence remained unstudied. The main scientific value of the present paper is that it reports on the efforts to explain why the raw material’s recycling may be harmful just for fatigue resistance. It must be emphasized that the explanation of the crystallographic induced mechanisms acting under powder’s recycling, being a logical continuation of the previously mentioned paper, constitutes a completely independent and novel research, which significantly contributes to the understanding of physics of possible recycling effect on the as-built and HIPed titanium alloys made product.

The presented study showed that in the process of phase recrystallization occurring during EBM and HIP, the macrotexture of Ti-6Al-4V-made parts is predominantly preserved and reproduces the texture of crystallization formed during primary melting. Phase transformations during repeated thermal cycling in EBM and heating/cooling during HIP occurred in accordance with the Burgers orientation relationship. This allows it to be stated that any phase transformations, including diffusion-controlled ones, include a pronounced shear component [40,44,45].

Repeated reuse of the powder in EBM leads to an increase in microhardness because of powder oxidation. Strengthening occurs due to the dissolution of oxygen in the α-phase as solid solution. The formation of the crystallographic texture in EBM-made parts does not lead to anisotropic mechanical properties. This study demonstrates that EBSD is an effective technique in investigation and even prediction of mechanical properties of titanium samples through microstructure evaluation and phases evolution analysis. The experimental findings also show that, for the parts that only need static strength and not fatigue, the severely recycled powder can be used in a combination with HIP treatment. Such approach pathway non-waste industrial additive manufacturing.

## Figures and Tables

**Table 1:** Selected recent publications on the same topic in comparison with the current review.

Ref	Powder Characterization	Powder Bed Fusion (PBF)		Powder Used for PBF		Effect of Powder Re-Use	HIP		EBSD Charact.
		EBM	SLM	New	Re-Used		Samples of New Powder	Samples of Re-Used Powder	
[13]	V	V	X	V	V	V	X	X	X
[14]	V	V	X	V	V	V	X	X	X
[16]	V	V	X	V	V	V	X	X	X
[17]	V	V	X	V	V	V	X	X	X
[18]	V	V	X	V	V	V	V	V	X
[20]	X	X	V	V	X	X	X	X	V
[21]	X	V	X	V	X	X	X	X	V
[23]	X	V	X	V	X	X	X	X	V
[24]	X	V	X	V	X	X	V	X	X
[25]	X	V	X	V	X	X	V	X	X
[28]	X	V	X	V	X	X	X	X	X
[29]	V	V	X	V	V	V	X	X	X
[30]	V	X	V	V	V	V	X	X	X
[31]	V	V	V	X	X	V	X	X	X
[32]	V	X	V	V	V	V	X	X	X
[33]	V	V	X	V	X	X	V	X	X
This work	V	V	X	V	V	V	V	V	V

**Table 2:** Arcam EBM process parameters.

Layer Thickness, μm	Alternating Angle between Layers	Beam Scan Speed, mm/s	Beam Power, W	Beam Focus Diameter, μm	Max. Beam Power, kW	Number of Contour Passes	Hatching Distance, μm
50	90	4530	1250	400	3	2	100

**Table 3:** Process parameters and microstructure characteristics of the samples.

Sample	Powder Used for EBM	Application of HIP	d_α_, μm	V_β_, %
S1	«virgin»	-	1.8 ± 0.3	3.3 ± 0.3
S2	«reused»	-	1.5 ± 0.3	1.7 ± 0.1
S3	«virgin»	+	2.5 ± 0.3	4.7 ± 0.3
S4	«reused»	+	2.7 ± 0.3	4.5 ± 0.3

**Table 4:** Oxygen content in powder, in as-built and treated samples (the data acquired from [18]).

Sample	“Virgin” powder	S1 sample—as-built from “virgin” powder	S3 sample—HIPed sample manufactured from “virgin” powder	ASTM req. %
Oxygen content, %	0.124	0.116	0.126	0.20 max
Sample	Re-used powder after 69th cycle	S1 sample—as-built from re-used powder	S3 sample—HIPed sample manufactured from re-used powder	
Oxygen content, %	0.324	0.336	0.350	

## Data Availability

The data presented in this study are available on request from the corresponding author. The data are not publicly available due to restrictions of the ongoing research project.
